# Blood pressure and renal cancer risk: the HUNT Study in Norway

**DOI:** 10.1038/sj.bjc.6603823

**Published:** 2007-05-22

**Authors:** L J Vatten, D Trichopoulos, J Holmen, T I L Nilsen

**Affiliations:** 1 Department of Public Health, Faculty of Medicine, Norwegian University of Science and Technology, Trondheim, Norway; 2 Department of Epidemiology, Harvard University School of Public Health, Boston, USA

**Keywords:** renal cell cancer, blood pressure, antihypertensive treatment, population study, epidemiology

## Abstract

In a prospective study of 36 728 women and 35 688 men during 18 years of follow-up, compared to systolic pressure <130 mm Hg, levels of 130–149, 150–169 and ⩾170 mm Hg in women were associated with relative risks of renal cell cancer of 1.7, 2.0 and 2.0, respectively (*P* for linear trend, 0.11). In men, there was no association with blood pressure.

The association of blood pressure with risk of renal cell cancer has been investigated in a number of cohort (Fraser *et al*, 1990; Grove *et al*, 1991; Coughlin *et al*, 1997; Heath *et al*, 1997; Chow *et al*, 2000; Choi *et al*, 2005; Flaherty *et al*, 2005; Fryzek *et al*, 2005; Lindgren *et al*, 2005; Schouten *et al*, 2005) and case–control (McLaughlin *et al*, 1995; Yuan *et al*, 1998; Shapiro *et al*, 1999) studies, using as principal exposure variable either recorded blood pressure or reported hypertension. In men, recorded blood pressure has shown a convincing exposure–response gradient related to renal cell cancer risk (Coughlin *et al*, 1997; Chow *et al*, 2000). In women, however, only reported history of hypertension has been studied as the main exposure variable. In one cohort (Flaherty *et al*, 2005) and two case–control studies (Yuan *et al*, 1998; Shapiro *et al*, 1999), history of hypertension was associated with increased risk. Documenting the presence or the lack of an exposure–response gradient in women is important, and might promote a better probing of the underlying mechanism of the association.

In this prospective study of 36 728 women and 35 688 men, we report on the association of blood pressure measured at baseline with renal cell cancer risk during 18 years of follow-up, together with that related to ever use of blood pressure medication.

## MATERIALS AND METHODS

In 1984, 85 100 individuals were invited to the Nord Trøndelag Health Study (the HUNT Study) in Norway, and 75 058 (88.2 percent) accepted the invitation, filled in a self-administered questionnaire, and attended a clinical examination (Holmen *et al*, 1991; Ellekjaer *et al*, 2000). Briefly, information included smoking status and standardised measurements of blood pressure, body height and weight. The study was approved by the Regional Committee for Ethics in medical research, and by the Norwegian Data Inspectorate.

This study was restricted to participants without prevalent cancer who had complete information on blood pressure and body mass index. Blood pressure was measured using calibrated mercury manometers with standard cuff size (Holmen *et al*, 1991). We divided systolic pressure (in mm Hg) into the following categories: <130 (reference), 130–149, 150–169 and ⩾170, and diastolic pressure (in mm Hg) into the following: <85 (reference), 85–94, 95–104 and ⩾105. Information on use of antihypertensive medication was derived from the question ‘do you use or have you ever used blood pressure medication?’

Body mass index was calculated as weight (in kg) divided by the squared value of height (in metres), and grouped into four categories: <18.5, 18.5–24.9, 25–29.9 and ⩾30. Information on smoking was categorised as never, former or currently smoking. Education was divided into three categories, depending on duration (<9, 10–12 and >12 years).

The mandatory reporting of cancer by physicians and hospitals to the Cancer Registry of Norway (www.kreftregisteret.no) provides information on incident cases of renal cell cancer that occurred during follow-up. Person-years were calculated from the clinical examination until the diagnosis of renal cell cancer or other cancers (except basal cell carcinoma), death from other causes, or the end of follow-up, 31 December 2002, whichever came first. The relative risk was calculated as the rate of renal cell cancer within a given blood pressure category compared with that in the reference category. We used Cox regression analysis to adjust for potential confounding by body mass index, smoking, use of blood pressure medication and education level. The statistical analyses were conducted using STATA, version 9.0 (StataCorp LP, 1985–2005).

## RESULTS

During 18 years of follow-up, 94 women and 144 men were diagnosed with renal cell cancer. Characteristics of the cohort are shown in Table 1. Risk was much lower in women with systolic pressure below 130 mm Hg than for those with higher levels, after adjustment for age, body mass index, smoking status, use of blood pressure medication and education (Table 2). Compared to the reference (<130 mm Hg), the adjusted relative risk in women with systolic pressure 130–149 mm Hg was 1.7 (95% confidence interval (CI), 0.9–3.5), with levels 150–169 mm Hg the risk was 2.0 (95% CI, 0.9–4.2), and with levels ⩾170 mm Hg, it was 2.0 (95% CI, 0.9–4.6). For diastolic pressure, the association with renal cancer risk was weaker than for systolic. Compared to the reference (<85 mm Hg), the adjusted relative risk in women with diastolic pressure ⩾105 mm Hg was 1.6 (95% CI, 0.8–3.5).

In a separate analysis restricted to people who reported never-use of blood pressure medication at baseline, there was a strong and positive association between systolic pressure and risk among women (Table 3). Thus, the adjusted relative risk in women with systolic pressure ⩾170 mm Hg compared with <130 mm Hg was 3.4 (95% CI, 1.3–8.9), and it showed a significant trend with increasing systolic pressure (*P* for trend, 0.001); diastolic pressure among never users of blood pressure medication, however, showed similar associations to the overall results.

Use of antihypertensive medication, as reported by women at baseline, showed a weak positive association (adjusted relative risk, 1.4, 95% CI, 0.8–2.2, Table 2). In men, there was no clear association with systolic or diastolic blood pressure (Tables 2 and 3), and none between blood pressure medication and risk (Table 2).

## DISCUSSION

The most striking finding in this study was that normotensive systolic blood pressure among women (<130 mm Hg) was associated with consistently lower risk for renal cell cancer than higher levels. No previous study of women has assessed the effect of measured blood pressure on this risk, but a positive association with recorded blood pressure has been found among men (Coughlin *et al*, 1997; Chow *et al*, 2000).

Correspondingly, studies of antihypertensive medication, or history of hypertension, have not shown consistent results. In two prospective studies, antihypertensive treatment was associated with higher risk of renal cell cancer (Fraser *et al*, 1990; Heath *et al*, 1997), but others found no association (Grove *et al*, 1991; Flaherty *et al*, 2005; Schouten *et al*, 2005). One large study linking prescriptions and cancer registration in Denmark indicated that antihypertensive medication may increase risk, but was interpreted as being due to confounding by underlying hypertension (Fryzek *et al*, 2005). One prospective study (Flaherty *et al*, 2005) and two case–control studies (Yuan *et al*, 1998; Shapiro *et al*, 1999) have also reported positive associations with a history of hypertension.

Contrary to previous evidence, we found that measured blood pressure, or use of blood pressure medication, was not associated with risk for renal cell cancer among men. The reason for this discrepancy is not obvious, but may be the play of chance.

In our study, systolic and diastolic blood pressures were measured according to standardised procedures by a team of trained nurses. The cohort consists of the majority of adults in a stable, homogeneous population in Norway, well suited for cancer follow-up, because of the national mandatory reporting system, and the unique identification number allocated to each citizen.

Angiogenic and other growth factors are associated with blood pressure increase, and may also be involved in the development of renal cell cancer (Schena *et al*, 1999; Chow *et al*, 2000; Choueiri *et al*, 2006). Thus, subtle long-term influences on renal function may lead to hypertension and also be related to tumour growth. The findings raise the possibility that high blood pressure may be a cause of renal cell cancer.

## Figures and Tables

**Table 1:** Characteristics of the study population (36 728 women and 35 688 men), stratified by systolic blood pressure categories

	**Women**				**Men**			
	**Systolic blood pressure**				**Systolic blood pressure**			
**Characteristic**	**<130**	**130–149**	**150–169**	**⩾170**	**<130**	**130–149**	**150–169**	**⩾170**
No. of participants	17 745	9251	5292	4440	12 209	14 333	5954	3192
No. of renal cell cancers	13	26	24	22	31	40	35	18
Median person years (range)	18 (19)	18 (19)	17 (19)	13 (19)	18 (19)	18 (19)	17 (19)	10 (19)
Mean age at baseline, years (s.d.)	39 (13)	52 (16)	63 (13)	70 (10)	42 (14)	46 (16)	57 (16)	67 (12)
Mean age at diagnosis, years (s.d.)	60 (15)	72 (12)	73 (10)	75 (9)	65 (12)	64 (14)	73 (9)	79 (8)
Body mass index, % ⩾25.0 kg m^−2^	25	52	64	70	37	51	62	62
Smoking status, % current	34	23	15	10	32	29	26	24
Educational level, % ⩾13 years	11	5	2	1	13	10	5	3

**Table 2:** Relative risk of renal cell cancer associated with standardised measured blood pressure and self reported blood pressure medication

	**Women**					**Men**				
**Variable**	**Person years**	**Cases**	**RR^a^**	**95% CI**	***P*_trend_ b**	**Person years**	**Cases**	**RR^a^**	**95% CI**	***P*_trend_ b**
*Systolic blood pressure (mm Hg)*										
<130	304 754	13	1.0	(Reference)	—	200 863	31	1.0	(Reference)	—
130–149	145 513	26	1.7	(0.9–3.5)	—	223 960	40	0.8	(0.5–1.4)	—
150–169	73 828	24	2.0	(0.9–4.2)	—	81 183	35	1.2	(0.7–2.0)	—
⩾170	53 060	22	2.0	(0.9–4.6)	0.11	33 890	18	1.0	(0.5–1.9)	0.59
										
*Diastolic blood pressure (mm Hg)*										
<85	346 610	31	1.0	(Reference)	—	264 778	41	1.0	(Reference)	—
85–94	145 246	22	0.8	(0.5–1.5)	—	173 219	56	1.6	(1.0–2.3)	—
95–104	64 349	22	1.4	(0.8–2.5)	—	76 975	20	1.0	(0.6–1.7)	—
⩾105	20 950	10	1.6	(0.8–3.5)	0.13	24 924	7	0.9	(0.4–2.0)	0.49
										
*Blood pressure medication* c										
No	504 135	57	1.0	(Reference)	—	499 677	106	1.0	(Reference)	—
Yes	73 020	28	1.4	(0.8–2.2)	—	40 219	18	1.1	(0.6–1.8)	—

CI=confidence interval, RR=relative risk.

a Adjusted for age, blood pressure medication (no, yes), body mass index (<18.5, 18.5–24.9, 25.0–29.9, ⩾30.0), smoking status (never, former, current, unknown), education (<10, 10–12, ⩾13 years, unknown).

b *P*-values from trend test using the categories as an ordinal variable in the Cox regression model.

c Adjusted for age, body mass index (<18.5, 18.5–24.9, 25.0–29.9, ⩾30.0), smoking status (never, former, current, unknown), education (<10, 10–12, ⩾13 years, unknown).

**Table 3:** Relative risk of renal cell cancer associated with standardised measured blood pressure among participants without blood pressure medication

	**Women**					**Men**				
**Variable**	**Person years**	**Cases**	**RR^a^**	**95% CI**	***P*_trend_ b**	**Person years**	**Cases**	**RR^a^**	**95% CI**	***P*_trend_ b**
*Systolic blood pressure (mm Hg)*										
<130	297 321	11	1.0	(Reference)	—	196 686	29	1.0	(Reference)	—
130–149	125 064	17	1.8	(0.8–4.1)	—	209 946	32	0.8	(0.5–1.3)	—
150–169	51 547	16	2.8	(1.2–6.7)	—	68 607	29	1.3	(0.7–2.2)	—
⩾170	30 202	13	3.4	(1.3–8.9)	0.005	24 437	16	1.3	(0.7–2.5)	0.22
										
Systolic blood pressure (per 10 mm Hg increase)	504 134	57	1.2	(1.1–1.3)	0.001	499 676	106	1.0	(1.0–1.2)	0.36
										
*Diastolic blood pressure (mm Hg)*										
<85	332 154	23	1.0	(Reference)	—	259 187	39	1.0	(Reference)	—
85–94	118 692	16	1.1	(0.6–2.1)	—	161 016	45	1.4	(0.9–2.2)	—
95–104	41 705	14	2.0	(1.0–4.0)	—	62 274	16	1.1	(0.6–1.9)	—
⩾105	11 583	4	1.7	(0.6–5.0)	0.08	17 199	6	1.2	(0.5–2.9)	0.11
										
Diastolic blood pressure (per 10 mm Hg increase)	504 134	57	1.2	(1.0–1.5)	0.13	499 676	106	1.1	(0.9–1.3)	0.56

CI=confidence interval, RR=relative risk.

a Adjusted for age, body mass index (<18.5, 18.5–24.9, 25.0–29.9, ⩾30.0), smoking status (never, former, current, unknown), education (<10, 10–12, ⩾13 years, unknown).

b *P*-values from trend test using the categories as an ordinal variable in the Cox regression model
